# Palliative Care in a Specialized Palliative Cancer Care Unit in Portugal: A Complex Reality

**DOI:** 10.7759/cureus.37930

**Published:** 2023-04-21

**Authors:** Beatriz R Sousa, Teresa Dias Moreira, Pedro Pires

**Affiliations:** 1 Internal Medicine, Centro Hospitalar Universitário de Lisboa Central, Lisbon, PRT; 2 Palliative Care, Instituto Português de Oncologia do Porto Francisco Gentil, Oporto, PRT

**Keywords:** patient care team, advanced cancer, interdisciplinary communication, palliative medicine, palliative care

## Abstract

Introduction

The goal of palliative care (PC) is to improve the quality of life of patients and their families through the involvement of a multidisciplinary team. PC improves symptom control and end-of-life care. Despite the fact that the benefits of PC have long been acknowledged, Portugal's demands are currently unmet. The majority of patients have been identified as having a high level of complexity and are referred for symptom management and end-of-life care.

Study aim

The study aimed to analyze the sociodemographic, disease and hospitalization characteristics of the patients admitted to a specialized PC unit.

Materials and methods

We conducted a retrospective, single-center study of palliative care patients admitted to a Portuguese oncology institute’s acute palliative care unit during a three-month period. Patients' information such as social demographics, clinical data, patient and family member’s psychological, social, nutritional and spiritual counseling and knowledge on diagnosis and therapy objectives were collected from physician’s records and analyzed using SPSS Statistics for Windows, Version 23.0 (IBM SPSS Statistics for Windows).

Results

A total of 41 patients were included, with a mean age of 66.4 years. Spouses were the primary caregivers. There was no indication for targeted therapy in any of the patients. Prior to hospitalization, 58.5% did not receive follow-up by PC. The most frequently reported symptoms were pain (75.6%), tiredness (68.3%), anorexia (61%) and emotional distress (58.5%). Patients were referred to counseling for psychological (43.3%), spiritual (19.5%), nutritional (58.5%) and social services (34.1%). During hospitalization, 75% of patients died; out of which, 70.9% were not previously followed up on by the PC team.

Conclusion

PC patients are complex, with multiple clinical-psychological-social-spiritual issues, and their management in non-PC wards can be challenging. Since the use of a multidisciplinary approach can improve patients’ and families’ quality of life, it is critical to train, expand and integrate the PC teams into the existing teams, allowing patients a better quality of life until they pass.

## Introduction

The goal of palliative care (PC) is to improve the quality of life of patients and their families who are dealing with the problems associated with a life-threatening illness by preventing and relieving physical, psychological, social or spiritual symptoms [1]. The PC teams are trained in four main areas: symptom management, communication, multidisciplinary teamwork and family support [2].

In 2020, there were 1,194 deaths per 100,000 people in Portugal, and it was estimated that 75% could have benefited from PC services [3,4]. The majority of patients in need of PC have chronic diseases such as cancer [5]. Cancer remains one of the leading causes of death in Portugal, accounting for 23.0% of all deaths in 2020 [6]. Cancer patients are 74% more likely to die in hospitals than patients with other diseases [7]. Although PC services in Portugal have steadily improved in recent years, more PC units for patients are still required; only 40-50 beds per 1,000,000 people are available in hospitals or National Integrated Continuing Care Network palliative care units (UCP), compared to the aim of 441 beds [4].

The vast majority of patients are referred to palliative care for symptom management and end-of-life care. At the end of life, the complexity factors increase in cancer patients, and the patients' needs become more complex [8]. The involvement of the PC team improves the quality of life in cancer patients [9]. However, many patients and relatives think that palliative care consultations are behind time, limiting the quality provided [10,11]. Information about hospitalization in a specialized palliative care unit is limited. In order to anticipate which patients to expect and to be knowledgeable about the essential skills of the PC teams as well as the current challenges, the purpose of this study is to analyze the sociodemographic, disease, and hospitalization characteristics of the patients admitted to a specialized PC unit.

## Materials and methods

Study design

The study was designed as a retrospective, single-center study conducted in a Portuguese oncology institute’s acute palliative care unit.

Patient selection

All admitted patients assigned to a single attending physician working group for a three-month period (July 1 to September 30, 2022) were included.

Data collection

Patients' sociodemographics information, such as age, gender, primary caregivers and religious orientation, and clinical data regarding primary cancer location, metastasis and past palliative care follow-up were collected from physician’s records, as well as dates of admission and discharge or death, provenance, reason for admission, symptoms and patient and family member’s psychological, social, nutritional and spiritual counseling; knowledge on diagnosis and therapy objectives was also collected. At the time of admission, the Eastern Cooperative Oncology Group (ECOG) performance status was used to assess functional status [12].

Data analysis

SPSS Statistics for Windows, Version 23.0 (IBM SPSS Statistics for Windows) was used for statistical analyses. Continuous variables such as age were reported as mean and standard deviation (SD). On the other hand, categorical variables such as qualitative data were presented as frequency and percentage.

## Results

During the three-month study, 41 patients were included for analysis. The average age was 66.4 ± 15.6 years (range, 39-89 years). About 63.4% were female. Due to delirium or an agonic state, 26.8% of the patients were unable to collaborate. The primary caregivers were mostly family members with spouses accounting for 36.6% of the total. Religious belief was reported by 46.4% of the total and 63.3% of those who were able to respond (Table 1).

**Table 1:** Epidemiological and social characteristics. SD, standard deviation; %, percentage; n, number of patients.

Variables	Mean (SD) or % (n)
Age	66.4 (15.6)
Gender	
Female	63.4 (26)
Male	36.6 (15)
Primary caregiver	
Spouse	36.6 (15)
Son or daughter	29.3 (12)
Formal caregiver	7.3 (3)
Other	14.6 (6)
Without caregiver	12.2 (5)
Religious beliefs	
Yes	46.4 (19)
No	26.8 (11)
Unknown	26.8 (11)

The most common primary cancer sites were genitourinary (19.5%) and head-and-neck (17%). On admission, 95.1% of patients had distant metastasis, with lymph nodes (53.7%) and hepatobiliary tract (26.8%) being the most common sites. At the time of the observation, none of the patients had indication for a cancer-directed treatment. Prior to hospitalization, 58.5% did not receive follow-up in PC. Of those who were able to collaborate, 86.7% were aware of the treatment’s non-curative goal. On admission, 53.7% of patients had ECOG performance status IV.

The patients were admitted to the hospital from the emergency department (56.1%), followed by intrahospital transfer from oncology units (22%). The reasons for admission were symptom control (68.3%) and end-of-life care (31.7%); social problems such as exhaustion or absence of a caregiver, as well as a lack of technical support, were identified in 36.8% of the patients, despite the fact that these were not the reason for hospitalization. Disease and hospitalization-related data are further detailed in Table 2.

**Table 2:** Disease and hospitalization-related data. %, percentage; n, number of patients; PC, palliative care; ECOG: Eastern Cooperative Oncology Group.

Variables	% (n)
Primary cancer sites	
Genitourinary	19.5 (8)
Head-and-neck	17 (7)
Lung	12.2 (5)
Stomach	12.2 (5)
Breast	9.8 (4)
Hepatobiliary tract	9.8 (4)
Colorectum	7.3 (3)
Others	12.2 (5)
Distant metastasis upon admission	
Yes	95.1 (39)
No	4.9 (2)
Main sites of distant metastasis	
Lymph node	53.7 (22)
Peritoneum	26.8 (11)
Lung	22 (9)
Bone	22 (9)
Hepatobiliary tract	19.5 (8)
Current cancer-directed treatment	
No	100 (41)
Knowledge of treatment’s non-curative goal	
Yes	63.4 (26)
No	9.8 (4)
Unknown	26.8 (11)
Previous follow-up with PC team	
Yes	41.5 (17)
No	58.5 (24)
Provenance	
Emergency department	56.1 (23)
Oncology units	22 (9)
Palliative care consultation	17 (7)
Home palliative care team	4.9 (2)
ECOG performance status upon admission	
1	7.3 (3)
2	14.6 (6)
3	24.4 (10)
4	53.7 (22)

Pain (75.6%), fatigue (68.3%), anorexia (61%) and emotional distress (58.5%) were the most frequently reported symptoms during hospitalization. Furthermore, some patients had delirium (36.6%), constipation (29.3%), dyspnea (26.8%), nausea (24.4%), malignant wound (19.5%) and xerostomia (17%). During hospitalization, the average number of problems managed per patient was five.

Patients were referred to counseling for psychological (43.3%), spiritual (19.5%, 42.1% of those with religious orientation), nutritional (58.5%) and social services (34.1%, all initially admitted for symptom control and 50% of those discharged). About 43.9% of family members were also referred to psychological counseling.

The average length of stay for all patients was 22.2 ± 28.3 days (range, 1-110 days). Seventy-five percent died in the hospital, with 70.9% not having previously been followed up on by the PC team; 32.3% died in the first week and 22.6% in the second. Ten (24.4%) patients were discharged, with nine (90%) of those returning home with social and technical support.

## Discussion

Almost all patients had distant metastasis, none had a target therapy indication, and the majority had not been followed up on by the PC team at the time of admission. Our patients’ late referral confirms previous concerns raised in other studies, with a late referral (< 90 days before death) being associated with increased duration of stay and odds of a patient dying in the hospital [13,14]. As soon as a clinical professional recognizes a patient with an advanced, progressive and life-limiting disease, articulation with the PC team should be made [15]. In our institute, referral to the PC team is almost always done when there is no longer an indication for disease-directed therapy. As described in other studies, the known causes for these are non-referral in earlier stages and a lack of human resources in the PC team [16]. Measures such as expanding the PC team and integrating into multidisciplinary decision groups are being implemented as recommended [16].

PC teams are trained to treat severe symptoms and manage end-of-life situations, which allows for better in-and-out hospital planning [17,18]. Because the majority of these patients had not previously been followed by the PC team, they were admitted through the emergency department, followed by intrahospital transfers from the oncology ward for symptom control and end-of-life care. Furthermore, the advanced stage of the disease resulted in severe functional limitations. These contribute to a better understanding of the complexity of PC patients and the difficulties in managing them outside of a PC unit, whether in a ward without trained professionals or at home [19,20].

On average, five problems were managed per patient, and the most common symptoms were similar to those reported in the literature [21,22]. A significant proportion of patients had physical, emotional or social problems, according to our findings. As the disease progresses, it is natural for physical symptoms to worsen, physical dependence to increase with loss of autonomy and emotional suffering to rise associated with loss of autonomy and concerns about the end of life. It is understandable that patients and their families are distressed, especially those who are aware of the treatment’s non-curative goal. Teams must be alert and intervene when suffering threatens the patients' quality of life. Psychological distress has been linked with greater physical symptom severity, suffering and mortality [23]. To address the emotional distress, a significant proportion of patients who were able to collaborate and relatives were referred to psychological and spiritual counseling. Although we did not assess the causes of emotional distress, one study found that financial difficulties, tiredness, work activity, pain and children were among the concerns mentioned [24]. As a result, a multidisciplinary team trained to recognize and respond to all types of suffering is essential.

Although 36.8% of patients had social problems upon admission, only patients admitted for symptomatic control were referred to social services, accounting for 34.1% of patients. The majority of the patients had an ECOG of IV, indicating that they are unable to perform self-care and spend the majority of their time in bed, which can be a physical and psychological source of distress for the family [25]. Social counseling helps patients and/or relatives maximize personal and community resources and integrate care [26]. Nutritional counseling was the most common type of counseling, with 58.5% of the patients referred to receiving it. Similar to the literature, the most common symptoms that could be improved with nutritional counseling in our population were anorexia, constipation, nausea and xerostomia [27]. Nutritional counseling, according to European Society for Clinical Nutrition and Metabolism (ESPEN) guidelines, is the first approach within a nutritional treatment, aimed at managing symptoms, encouraging the intake of better-tolerated foods and beverages, and managing preferences [28].

When compared to the number of patients initially admitted for end-of-life care, the mortality rate was high, with more than 50% of patients dying within the first two weeks. This finding is consistent with previous research [29]. Almost 80% of the patients had never been followed up on by the PC team. A short time between referral and death indicates a missed opportunity for a multidisciplinary team specializing in PC to intervene early to alleviate the physical, emotional, social and spiritual suffering of patients and their families.

There were several limitations in this study. It was a retrospective study carried out in a short period of time with patients from a single group of work, rather than all patients admitted to the palliative care service, in a single institution. It is a palliative care unit at a cancer institute, staffed by a well-established PC team, with a lack of patients suffering from diseases other than cancer.

## Conclusions

Palliative care patients are complex, with multiple clinical-psychological-social-spiritual issues. This management can be difficult in other medical wards that are not devoted to PC patients. The delay in the referral and the fragmentation of services withhold the opportunity to manage these components in a timely manner to mitigate the suffering of those we care for. Using a multidisciplinary approach, specialized PC units can identify and relieve clinical, psychological, social and spiritual symptoms presented by patients and relatives, thereby improving quality of life. It is critical to raise awareness of these issues among healthcare professionals, train professionals in this field in order to expand the teams, form specialized units and integrate PC teams into existing teams, allowing patients to live with quality of life. More thoroughly planned research are required to comprehend the difficulties and identify the needs of individuals requiring palliative care.
